# Effect of the Compatibiliser on the Poly(Lactic Acid)—Polyamide 11 Blends with and Without Metal Oxides: Properties, Performance and Durability

**DOI:** 10.3390/polym18141782

**Published:** 2026-07-21

**Authors:** Giulia Infurna, Federico Ferrante, Elisabetta Morici, Giuseppe Pecoraro, Nadka Tz. Dintcheva

**Affiliations:** 1 Dipartimento di Ingegneria, Università di Palermo, Viale delle Scienze, Ed. 6, 90128 Palermo, Italy; giulia.infurna@unipa.it (G.I.); federico.ferrante01@community.unipa.it (F.F.); elisabetta.morici@unipa.it (E.M.); giuseppe.pecoraro09@community.unipa.it (G.P.); 2 ATEN Center, Università di Palermo, Viale delle Scienze, Ed. 18, 90128 Palermo, Italy; 3 Consorzio Interuniversitario Nazionale di Scienza e Tecnologia dei Materiali (INSTM), Via Giusti, 9, 50121 Firenze, Italy

**Keywords:** biopolymer blends, compatibiliser, metal oxides, properties and performance, durability

## Abstract

It can be argued that blends of biopolymers can be regarded as an economical and efficacious method for formulating blends with customised properties. In the context of immiscible and/or incompatible constituents, the use of a compatibiliser agent (i.e., physical or chemical compatibiliser) has been demonstrated to enhance specific properties, including ductility and hydrophobicity. In this study, biopolymer blends based on polylactic acid (PLA) and polyamide 11 (PA11), with and without a compatibiliser (ethylene butyl-acrylate glycidyl methacrylate; Elvaloy), and also in the presence of metal oxides, such as zinc oxide (ZnO) and titanium dioxide (TiO_2_), were processed by melt mixing and characterised for their mechanical, rheological, thermal and hydrophobic behaviour, as well as for their photo-oxidation resistance. The presence of a compatibiliser appeared to have a beneficial effect on the system’s ductility and hydrophobicity, also changing the blend morphology and reducing the dimensions of the PA11-droplets. The complex nature of the systems in question means that the beneficial effect of the compatibiliser on the dispersion of metal oxides cannot be fully appreciated. The favourable dispersion of metal oxide particles, in conjunction with their selective location at the interphase between the two polymeric phases and/or in the more polar phase, reduces the photo-oxidation resistance of these systems. This aspect must be given due consideration.

## 1. Introduction

Changing from fossil-based plastics to bio-based polymers solves several problems associated with the production and use of conventional plastics. Specifically, there are several advantages to using bio-based polymers: (i) Reduction of dependence on finite resources: most fossil-based polymers come from petroleum or natural gas, which are limited resources located in geopolitically confined zones. Bio-based polymers, on the other hand, are derived from renewable feedstocks such as plants (e.g., corn, sugarcane or castor oil), which can be replenished within a human lifetime, and even better if the resources come from plant waste. (ii) Lower carbon footprint: when producing biopolymers such as polylactic acid (PLA), polybutadiene succinate (PBS), polybutadiene adipate-co-terephthalate (PBAT), polyamide 11 (PA11) or polyglycolic acid (PGA), the carbon in the polymer originally comes from atmospheric CO_2_ captured by plants via photosynthesis. Overall, this can offset emissions to a certain extent compared to fossil plastics, which introduce “new” carbon dioxide into the atmosphere. (iii) Support for circular economy strategies: some bio-based polymers are biodegradable or compostable under the right conditions. This can help reduce long-term plastic accumulation, particularly in applications such as packaging. Even when the polymers are not biodegradable, they can be incorporated into recycling or bio-refinery systems. In summary, shifting from fossil-based to bio-based polymers is beneficial because it shifts materials production towards renewable carbon, which could reduce environmental impact and align plastics with long-term sustainability goals [1,2,3,4,5,6,7,8].

Biopolymer blends, such as PLA-PBAT [9], PLA-PA11 [10,11,12,13,14], PLA-PGA [15], PLA-thermoplastic starch [16,17], PBAT-thermoplastic starch [18], etc., are advantageous in that they bridge the gap between biodegradable brittle plastics and durable engineering polymers, enabling applications such as packaging, automotive parts and 3D-printed components where neither material alone performs well. Therefore, the blending of biopolymers has emerged as a highly effective and economically viable strategy [19,20]. The physical combination of two or more polymers has been shown to result in a tailoring of mechanical, thermal, rheological and barrier properties, obviating the necessity for complex chemical synthesis. The design of biopolymer blends is intended to achieve a balance between stiffness and toughness, to improve impact resistance, to enhance biodegradation behaviour and to reduce overall material cost. In many cases, blending also allows the incorporation of biodegradable polymers into existing industrial processing routes, facilitating large-scale adoption [9,10,11,12,13,14,15,16,17,18,19,20]. Overall, considering their constituents, composition, processability and performance, the biopolymer blends can be considered useful for various application fields, such as food packaging [16,17,18,21], the biomedical and pharmaceutical sector [21,22,23,24], the automotive industry [25] and environmental/water remediations [26,27,28]. Another important consideration in the formulation of blends is the recyclability of biopolymers at the end of an item’s life. Currently, however, biopolymers are not separated from fossil-based polymers, which causes numerous problems in recycling processes [29,30,31].

The formulation of biopolymer-based blends, nanocomposites using naturally occurring fibres and fillers, nano-clay, metal oxides, etc., opens new windows toward the development of new polymeric materials with desired properties with significantly reduced environmental impacts. The biodegradable blend nanocomposites can perform smart applications in industrial, pharmaceutical and biomedical fields by tailoring their properties, and, as is known, the properties of blends can be enhanced by the addition of nanofillers with or without modifications [32,33,34,35,36,37,38]. The presence of nanometric particles in polymers and biopolymers has been shown to have the potential to be harmful if the particles are not well incorporated into the polymer or biopolymer matrices. However, migration of the particles is not favoured, and the nanoparticles have been shown to have beneficial effects on the composite performance. It is therefore hypothesised that there may be a negative effect on the end-of-life disposal of micro-/nano-composites. This highlights the need for accurate separation and management of these materials. The recycling of micro-/nano-composite materials necessitates their separation from poor polymer or biopolymer waste, due to the incompatibility of the materials [38].

The incorporation of metal oxide nanoparticles could have a beneficial effect on the composite’s physicochemical properties, such as mechanical, thermal, biological, electrical, optical and surface properties. The most commonly used metal oxide nanoparticles as suitable additives for biopolymers/polymers are zinc oxide (ZnO), titanium dioxide (TiO_2_), silver oxide (Ag_2_O), tin dioxide (SnO_2_), copper oxide (CuO), etc., although their dispersion in melt matrices is a difficult matter [39,40,41,42,43,44,45,46,47]. As is known, zinc oxide (ZnO) is chemically accessible and low-cost with a high redox potential able to remove organic pollutants, and acts as an anti-bacterial agent [13,14,39,40,41]. Titanium dioxide (TiO_2_) possesses numerous advantageous properties, such as stable structure, non-toxicity, anti-corrosion properties and high photocatalytic activity [13,14,42,43]. Both ZnO and TiO_2_ exhibit photocatalytic activity because of a positive band position that generates holes (h+) and electrons (e−) by molecular excitation under UV light, resulting in the generation of hydroxyl radicals and reactive oxygen species, which, in turn, can interfere with pollutants and bacterial metabolism, inhibiting their growth or causing bacterial death [42,43]. Other metal oxides, such as silver oxide (Ag_2_O) [44], tin dioxide (SnO_2_) [45] and copper oxide (CuO) [46,47], are usually proposed as modifiers of the structural, optical, electrical and antibacterial properties of biopolymers/polymers with potential applications in the packaging sector.

Interestingly, as documented in previous studies, the introduction of approximately 30 wt% PA11 into the PLA matrix facilitates the formulation of bio-based blends with adequate processability, mechanical performance, heat resistance and durability [12]. Although PLA and PA11 are immiscible, they can be organised from PA11-droplet morphology up to a continuous one, which leads to a significant improvement of the blend performance. Furthermore, the morphology of the biopolymer blends can be manipulated and refined using nanoparticles, such as nano-clay and metal oxides, which can be located at the interface between the two polymeric phases or within the more polar polyamide phase [12,13,14]. Therefore, as documented, the introduction of metal oxides, such as zinc oxide or titanium dioxide at low amounts, e.g., from 0.5 to 1 wt%, can modify the blend’s morphology and improve the mechanical performance, slightly penalising the blends’ photo-oxidation resistance [12,13]. To improve the compatibility of the metal oxides with the host matrix and to tailor nanoparticle dispersion, the particles can be chemically modified, for example, through the introduction of short alkyl chains and/or ultrasound treatment, also reducing particle agglomerations and improving the dispersion into the high-viscosity matrix during processing [14]. In summary, in our previous works [12,13,14], PA11 has been added to PLA at different ratios (i.e., 85/15, 70/30 and 50/50 wt%) in order to identify the optimal composition for fully bio-based blends with improved thermo-mechanical performance. Furthermore, the effect of the addition of reduced amounts of ZnO and TiO_2_ (at 0.5, 1 and 2 wt%) has also been investigated, considering the functional properties of these two metal oxides (e.g., antimicrobial, catalytic properties, etc.). It has also been demonstrated that surface modification of ZnO through the use of stearic acid or the ultrasound treatment of TiO_2_ results in enhanced dispersion of these metal oxide nanoparticles in the fully bio-based PLA/PA11 blends. The presence of stearic acid-modified ZnO and ultrasound-treated TiO_2_ has been demonstrated to induce morphological variations through their selective location in the more polar phase (i.e., PA11) or at the interface between the two polymer phases. However, these modifications have not been shown to yield significant improvements in blend properties and performance.

Although applying the processing-driven approach, i.e., the use of organo-modified nanoparticles that can modify and tune the processability and/or morphology, it is not possible to obtain optimal compatibilisation, and the use of a chemical compatibiliser is required. Taking this into consideration and based on our previous experiences, in this work, biopolymer blends based on polylactic acid (PLA) and polyamide 11 (PA11) with and without a compatibiliser, such as an ethylene butyl-acrylate glycidyl methacrylate (named Elvaloy), have been introduced at 5 wt%. To explore the effect of the compatibiliser on the mechanical, thermal, rheological and morphological properties, as well as on photo-oxidation resistance, different blend ratios have been processed, specifically PLA/PA11/Elvaloy at 85/15/5, 70/30/5 and 50/50/5 wt%, and compared with blends without a compatibiliser. Furthermore, to investigate the effect of the compatibiliser on the metal oxides dispersion, processed blends PLA/PA11/Elvaloy = 70/30/5 wt% with ZnO and TiO_2_ were used, and their performance was compared with the performance of blends without the compatibiliser. The findings of this study demonstrate that the compatibiliser assists in the dispersion of metal oxide particles and decreases the PA11 droplet dimensions. This effect is more evident in zinc oxide than in titanium dioxide.

## 2. Materials and Methods

### 2.1. Materials

The polylactic acid (PLA) used in this work was a commercial extrusion sheet grade supplied by NatureWorks (Blair, NE, USA, named PLA 2002D) with an average molecular weight of about 121,000 g/mol, a ratio of 96% L-lactide to 4% D-lactide units and a melt flow index of 6 g/10 min (230 °C, 2.16 Kg).

The polyamide used in this work was a Polyamide 11, PA11, (Nylon 11, pellet form, from Sigma-Aldrich (Taufkirchen, Germany)), with glass transition temperature Tg = 46 °C, melting temperature Tm = 198 °C and density ρ = 1.026 g/cm^3^ at 25 °C; molecular weight Mw = 201.31 g/mol, MFI@235 °C/2.16 kg = 14.5 ± 1.2 g/10 min.

Elvaloy^®^PTW is a terpolymer of ethylene, butyl acrylate and glycidyl methacrylate (EBAGMA) purchased from DuPont (Wilmington, DE, USA) in pellet form. EBAGMA is derived from 66.75 wt%. ethylene, 28 wt% n-butyl acrylate and 5.25 wt% glycidyl methacrylate. According to the manufacturer, the main physical properties of Elvaloy^®^PTW are an MFI of 12 g per 10 min, a melting point of 72 °C, a glass transition temperature of −55 °C, a density of 0.94 g/cm^3^, a tensile stress at break of 5 MPa and an elongation at break of 950%.

Zinc oxide (ZnO) nanopowder (<100 nm particle size) was purchased from Sigma-Aldrich and used without any further purification. According to previous investigations, ZnO nanoparticle size spanned a 100–200 nm range, with a mix of tubular and round-shaped forms [38].

Titanium dioxide (TiO_2_, AEROXIDE^®^ TiO_2_ P25) was purchased from Evonic and used without further purification. Its features are specific surface area (BET): 35–65 m^2^/g; tamped density: approximately 140 g/L, loss on drying < 1.5%, pH-value 3.5–4.5 and SiO_2_ content < 0.2%.

### 2.2. Melt Blending

The biopolymers (PLA and PA11), compatibiliser (Elvaloy) and metal oxides (ZnO and TiO_2_) were vacuum-dried at 80 °C overnight before processing to minimise hydrolytic degradation during melt processing. Blends PLA/PA11 = 85/15, 70/30, 50/50 wt% (named PLA85, PLA70 and PLA50, respectively), and blends containing compatibiliser at 5 wt% PLA/PA11/Elvaloy = 85/15/5, 70/30/5, 50/50/5 wt% (named PLA85/E, PLA70/E and PLA50/E, respectively) and metal oxides (both ZnO and TiO_2_ at 1 wt%) were prepared by melt mixing using a Brabender PLA-330 internal mixer at 200 °C for 5 min at 50 rpm. To improve the dispersion of both ZnO and TiO_2_ in PLA/PA11 and PLA/PA11/Elvaloy blend melt, the biopolymers were pre-mixed for 1 min, and then the metal oxides were added; the mixing was continued for a further period of 4 min.

The PLA/PA11/Elvaloy weight ratios of 85/15/5, 70/30/5, and 50/50/5 were selected to preserve the relative proportions of PLA and PA11 within the blend and to include the incorporation of additives. Thin films, with a thickness of approximately 100 microns, were obtained through the hot compression moulding process for the purpose of characterisation.

### 2.3. Characterisations

#### 2.3.1. Tensile Test

Tensile tests were carried out using a universal testing machine (Instron model 3365, High Wycombe, UK), according to the ASTM D882 method, on rectangular samples. The tests were performed using a tensile speed of 1 mm/min for 1 min to evaluate Young’s modulus, and then the velocity was increased to 10 mm/min until sample breakage. The tensile test was performed on ten valid samples of each material.

#### 2.3.2. Rheological Analysis

Rheological tests were performed using a stress-controlled rheometer (ARES G-2) in parallel plate geometry (plate diameter 25 mm). The complex viscosity (η*), storage (G′) and loss (G″) moduli were measured under frequency scans from ω = 0.1 to 100 rad/s at T = 170 °C. The strain amplitude was γ = 5%, which preliminary strain sweep experiments proved to be low enough to be in the linear viscoelastic regime.

#### 2.3.3. Scanning Electron Microscopy (SEM)

The morphologies of the blend nanocomposites were investigated by scanning electron microscopy images (SEM, Gemini 152 field emission SEM Supra 25, Carl Zeiss, Oberkochen, Germany). The images were obtained in the Inlens mode at 5 kV. The samples of neat PLA and PLA/PA11 were cryogenically fractured in liquid nitrogen to obtain a cross-section and subsequently sputter-coated with gold (10 mA, 4 min) to create a conductive surface layer of 10 nm.

The average numerical diameter (dn), Equation (1), average volumetric diameter (dv), Equation (2), dispersion (D), Equation (3) and the diameters of the dispersed phase were determined by measurements on the obtained SEM images, containing more than one hundred dispersed phase particles (ni PA11 particles, having di diameters) per sample, according to the following equations:

(1) $$d_{n}=\frac{\Sigma_{i}n_{i}d_{i}}{\Sigma_{i}n_{i}}$$

(2) $$d_{v}=\frac{\Sigma_{i}n_{i}d_{i}^{4}}{\Sigma_{i}n_{i}d_{i}^{3}}$$

(3) $$D=\frac{d_{v}}{d_{n}}$$

For particles having an elliptical form, an equivalent diameter, Equation (4), was used through the particle area, according to the following equation:

(4) $$d_{eq}=2{\left(\frac{A}{\pi }\right)}^{\frac{1}{2}}$$

where π = 3.14 and A is the surface.

#### 2.3.4. Water Contact Angle (WCA)

The water contact angle (WCA) was measured at room temperature using First Ten Angstrom (USA) FTA1000C system (Data Physics Instruments, Filderstadt, Germany), with demineralised water. The films were fixed on top of a solid plane support and kept flat during water deposition and acquisition. The sessile drop method was used with a droplet volume of 6 μL.

#### 2.3.5. Differential Scanning Calorimetry (DSC)

The calorimetric analysis was evaluated by differential scanning calorimetry (DSC), using a TA Instruments Q100 in a nitrogen atmosphere on 5 ± 0.5 mg samples sealed in aluminium crucibles. Samples were heated from ambient temperature to 230 °C at 10 °C/min, held in equilibrium conditions for 3 min at 230 °C to eliminate the thermal history, cooled to 25 °C at 10 °C/min and then reheated to 230 °C at 10 °C/min.

#### 2.3.6. Spectroscopy Characterisation

A Fourier Transform Infrared Spectrometer (Spectrum One, Perkin Elmer, Shelton, CT, USA) was used to record IR spectra using 16 scans at a resolution of 1 cm^−1^. The progress of photo-oxidation degradation of the samples was followed by FTIR analysis, monitoring the variations of carbonyl range (1850–1600 cm^−1^) in time, using Spectrum One software.

### 2.4. Accelerated Photo-Oxidation Exposure

Accelerated photo-oxidation was carried out using a Q-UV/basic weatherometer (from Q-LAB, Westlake, OH, USA) equipped with UVB lamps (313 nm). The simulated weather conditions consist of continuous light irradiation at T = 70 °C.

The progress of the photo-oxidation of PLA/PA11 = 70/30 wt./wt% and blends containing metal oxides was monitored by FTIR analysis at different exposure times, following the height of the peak at 1845 cm^−1^, assigned to the accumulation of the anhydride functionalities in the PLA phase due to the PLA photo-oxidation, and the height of the peak at 1725 cm^−1^, assigned to the accumulation of carbonyl functions in the PA11 phase due to PA11 photo-oxidation [14]. The spectra were not normalised because it is impossible to correctly identify an appropriate peak for normalisation in the blend systems.

## 3. Results

### 3.1. Effect of Compatibiliser on Biopolymer Blends

Biopolymer blends often experience problems such as immiscibility, poor interfacial adhesion and phase separation, and to improve compatibility between the constituents and enhance chain interdiffusion, compatibilisers can be incorporated, which can substantially improve the overall performance of the blend. PLA/PA11 blends at 85/15, 70/30 and 50/50 wt%, with and without a compatibiliser (Elvaloy) added at 5 wt%, were characterised for their mechanical, rheological and morphological behaviour, as well as for their photo-oxidative resistance. The selection of compatibiliser, for example, a terpolymer of ethylene, butyl acrylate and glycidyl methacrylate, has been made with consideration for the design of blends that have the potential to be used for food packaging applications and are free of maleic anhydride modified polymers. Moreover, according to the literature, the compatibiliser must induce morphological change while not being present in excessive quantities, in order to preserve the macroscopic properties [48,49]. Taking this into account, the compatibiliser content for this work has been selected to exclude the introduction of a substantial amount of a constituent that could compromise performance.

**Figure 1:**
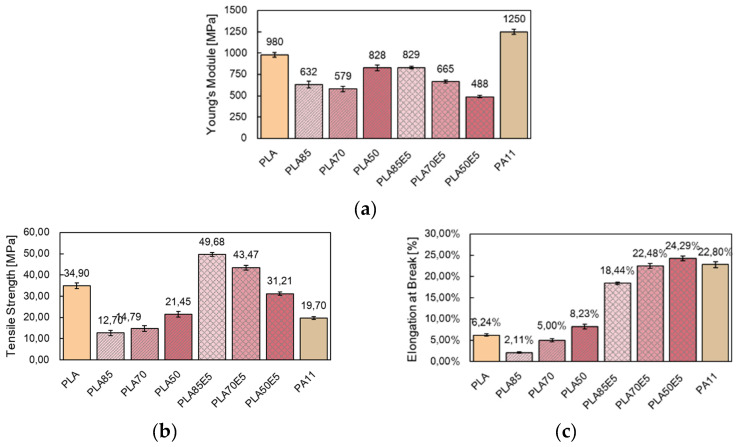
Mechanical properties: (**a**) Young’s modulus, E, (**b**) tensile strength, TS and (**c**) elongation at break, EB, of PLA/PA11 blends at 85/15, 70/30 and 50/50 wt% with and without compatibiliser agent (Elvaloy) at 5 wt%. Data on PLA/PA11 blends without compatibiliser have been published previously [13].

Figure 1a–c reports the main mechanical properties, i.e., (a) Young’s modulus, (b) tensile strength and (c) elongation at break. According to the literature, neat PA11 exhibits a higher Young’s modulus, indicative of greater rigidity, in comparison to neat PLA, and this phenomenon can be attributed to the presence of intramolecular hydrogen bonding in amide groups in both the crystalline and amorphous phases [50]. As was also documented in previous investigations, the PLA/PA11 blends without a compatibiliser (at 85/15, 70/30 and 50/50 wt%) exhibit mechanical properties intermediate between those of the two neat polymers and they are significantly fragile [12,13,44]. The beneficial effect of the compatibiliser presence on blend rigidity, i.e., Young’s modulus values, is evident in the 85/15/5 and 70/30/5 blends, while the opposite effect is noticed in the 50/50/5 blend, see Figure 1a. It can be seen that blends containing a compatibiliser exhibit increased breaking properties, i.e., both tensile strength and elongation at break values are higher for compatibilised blends than for blends without a compatibiliser (see Figure 1b,c). As expected, the compatibiliser presence plays a positive role in the ductility of the biopolymer blends. The results for PLA/PA11 without compatibiliser indicate the total absence of compatibility, thereby suggesting that the interface is incapable of effectively transferring stress between the biopolymers. This deficiency results in the formation of local stresses, which in turn lead to the development of voids. It is noteworthy that blends of PLA/PA11/E, i.e., with compatibiliser, demonstrate enhanced efficacy in transferring tensile stress and exhibit favourable morphology, as detailed below.

**Figure 2:**
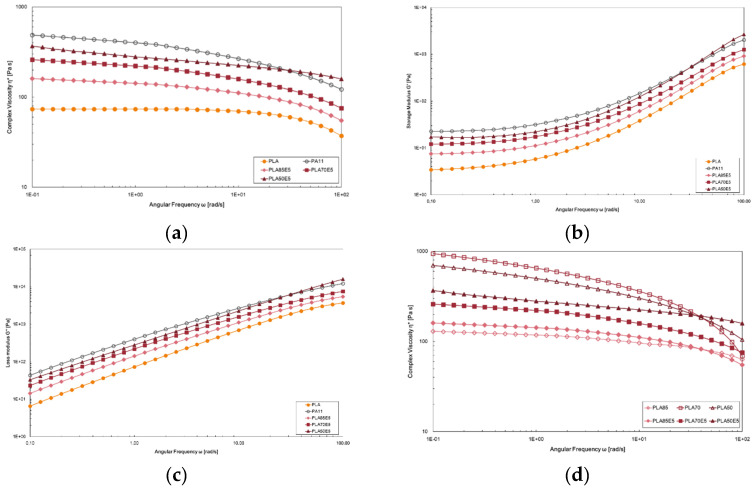
(**a**) Complex viscosity, (**b**) storage modulus, G′, (**c**) loss modulus, G″, of neat PLA, neat PA11 and PLA/PA11 blends at 85/15, 70/30 and 50/50 wt% with compatibiliser agent (Elvaloy PTW) at 5 wt%, and (**d**) comparison of viscosity trends for PLA/PA11 (open symbols) and PLA/PA11/Elvaloy (full symbols). Data on PLA/PA11 blends without compatibiliser have been published previously [13].

The rheological behaviour of the blends, as well as the behaviour of the neat PLA and PA11, has been evaluated by an oscillatory test. The trends of viscosity, storage (G′) and loss (G″) moduli are shown in Figure 2a–c. Moreover, Figure 2d presents a comparison of viscosity trends for blends with compatibiliser (full symbols) and without compatibiliser (open symbols). The rheological behaviour of neat PLA and neat PA11 is found to be significantly divergent; as can be seen, PLA manifests Newtonian behaviour across the entire frequency range, and conversely, the neat PA11 exhibits higher values of the complex viscosity related to the intermolecular interactions and well-pronounced shear-tinning behaviour at high frequencies. The PLA/PA11/E blends display viscosity trends intermediate between the two neat biopolymers, and the viscosity values increase with increasing PA11 amount. At low amounts of PA11, the rheological behaviour is governed by the PLA, and the viscosity, although slightly increases with compatibiliser presence. For the blends PLA/PA11/E at 70/30/5 and 50/50/5 wt%, it seems that the compatibiliser causes a viscosity decrease, due to the formation of smaller and well-dispersed PA11 drops.

**Figure 3:**
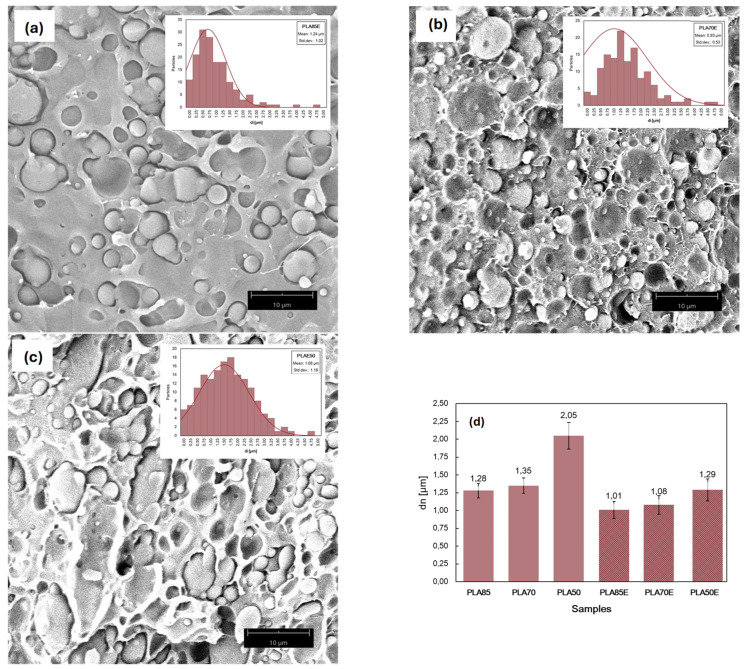
SEM observations and distribution of PA11 particle dimensions of PLA/PA11/E blends at (**a**) 85/15/5, (**b**) 70/30/5, (**c**) 50/50/5 wt%. (**d**) Average diameter (dn) of PA11 particles in PLA/PA11 and PLA/PA11/E blends. Average diameter (dn) data on PLA/PA11 blends without compatibiliser have been published previously [13].

In Figure 3a–c, SEM micrographs of PLA/PA11/E blends are presented at the same magnifications, and the statistical distributions of the dispersed phase size (i.e., PA11 particles) are reported as inserts. Furthermore, in Figure 3d, the average diameters (dn) of PA11 particles in PLA/PA11/E, in comparison to the data of PLA/PA11 blends, are reported. The SEM analysis provides a reliable representation of the droplet dimensions of the PA11 phase, and the dispersion of metal oxide nanoparticles has been deduced indirectly, based on the data. Drop-matrix morphologies manifest as a continuous PLA matrix and PA11 droplets, exhibiting diverse dimensions and shapes. As demonstrated in the extant literature, the morphology of PLA/PA11 in the absence of a compatibiliser (not presented here) appears to have two distinct biopolymer phases. The presence of the compatibiliser agent, despite the theoretical miscibility of PLA/PA11, is primarily associated with the respective low and high polarity of the two biopolymers. Therefore, the presence of small voids at the PLA/PA11/E interface can also be observed, and as expected, the voids in the morphology of PLA/PA11/E blends are significantly smaller than the voids in the morphology of PLA/PA11 blends. Therefore, the interface region could be considered the weakest point in the blend, where failure leads to fracture occurrences. The presence of a compatibiliser modifies the average diameter of the PA11 droplet particles, see Figure 3d; and in Table 1, the average diameter (dn) of PA11 particles in PLA/PA11 and PLA/PA11/E and the ratio (D) between dn and dv in the blends are reported. During the processing of polymer blends, dispersed droplets undergo breakups and coalescence, which are two concurrent processes. Furthermore, at high amounts of PA11, droplet coalescence phenomena dominate in the PLA, and the shape of the droplet’s changes from circular to elliptical. It is worth noting that the compatibiliser can reduce the average diameter of the dispersed PA11 phase and that the extension of the interface region decreases in comparison to a blend without a compatibiliser. The presence of a compatibiliser minimises the void formation, thereby improving adhesion between the PLA and PA11, and for this reason, PLA/PA11/E blends appear less fragile than PLA/PA11 blends.

However, despite the correlation between rheological and morphological analyses, it is crucial to recognise the necessity of analyses of the melt and solid state, respectively. The rheological analysis is indicative of the processability, i.e., the flow ability, of the blends, while the morphological analysis is also influenced by the solidification process and the selective location of the nanoparticles.

**Figure 4:**
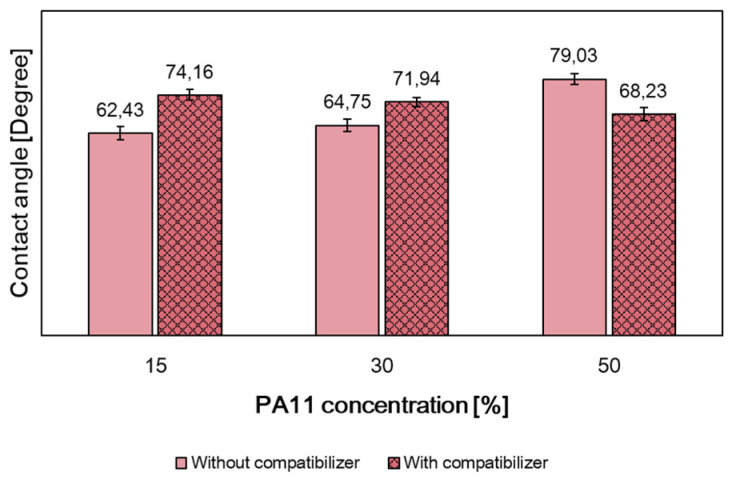
Water contact angle (WCA) measurements of PLA/PA11 with and without compatibiliser. Data on PLA/PA11 blends without compatibiliser have been published previously [13].

Surface analysis was conducted through the measurement of water contact angle (WCA), and in Figure 4, the WCA values of PLA/PA11 blends, with and without the addition of a compatibiliser, are reported. It has been demonstrated that the presence of the compatibiliser results in a slight increase in the surface hydrophilicity of PLA-based blends (i.e., PLA/PA11/E = 85/15/5 and 70/30/5 wt%). The 50/50/5 wt% blend of PLA/PA11/E exhibited a decline in WCA value due to the presence of compatibiliser in comparison with the blend of PLA/PA11, which can be attributed to an augmented system heterogeneity and complexity. It is worth noting that the films have been processed under the same processing conditions, and the changes in WCA values could be attributed to the blend compositions, i.e., to the presence of the compatibiliser.

The thermal behaviour was investigated using differential scanning calorimetry analysis. Table 2 summarises the results obtained for PLA/PA11 = 70/30 wt% with and without a compatibiliser, and figures in the Supplementary Information (Figures S1–S3) show the heating and cooling scans. The presence of the compatibiliser does not have a clearly pronounced effect on the thermal behaviours, probably because of the system complexity, i.e., the presence of different constituents, and the effect of any single constituent is difficult to identify.

According to the literature, the photodegradation process of PLA mainly involves chain scission, resulting in the formation of anhydride functionalities. This leads to the formation of a shoulder at 1845 cm^−1^, located close to the large peak of the carbonyl range (i.e., 1800–1700 cm^−1^), due to the presence of intrinsic carbonyl functionalities [51,52]. Furthermore, the photochemical oxidation of pure PLA and PLA-based blends or nanocomposites can be effectively monitored by observing the change in the shoulder/peak at 1845 cm^−1^. Specifically, according to our previous publications, the photo-oxidation of PLA/PA11 blends with and without metal oxides was monitored by observing both the shoulder/peak at 1845 cm^−1^ and the peak at 1725 cm^−1^, which is attributed to the formation of novel oxygen-containing species (e.g., carbonyl and carboxyl functionalities) in PA11 phase [53,54]. As previously outlined, PLA is a durable polymer that degrades when exposed to UV irradiation, requiring a longer exposure time than PA11 for degradation. As demonstrated in [44], both polymers photodegrade in the presence of oxygen via H-abstraction and the occurrence of Norrish I and II reactions.

**Figure 5:**
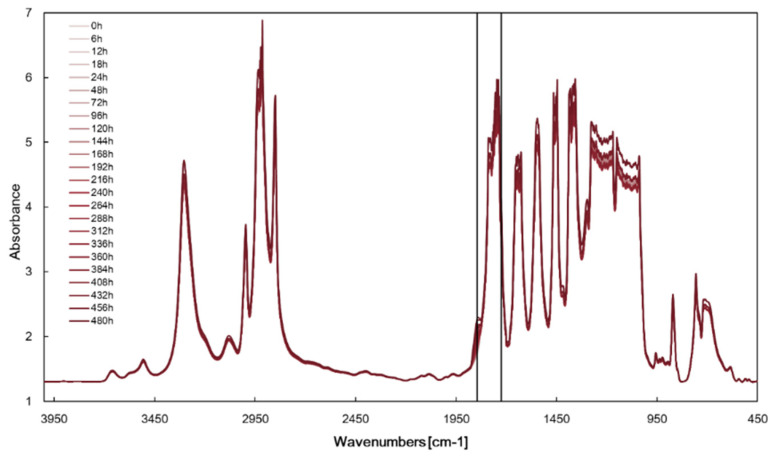
FTIR spectra of PLA/PA11/E = 70/30/5 wt% at different photo-oxidation times.

**Figure 6:**
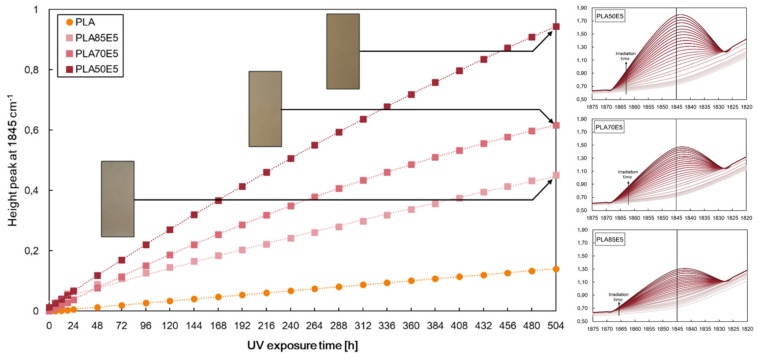
Height of peak at 1845 cm^−1^ as a function of the photo-oxidation time and selected zone in FTIR spectra of PLA/PA11/E blends at 85/15/5, 70/30/5, 50/50/5 wt%.

**Figure 7:**
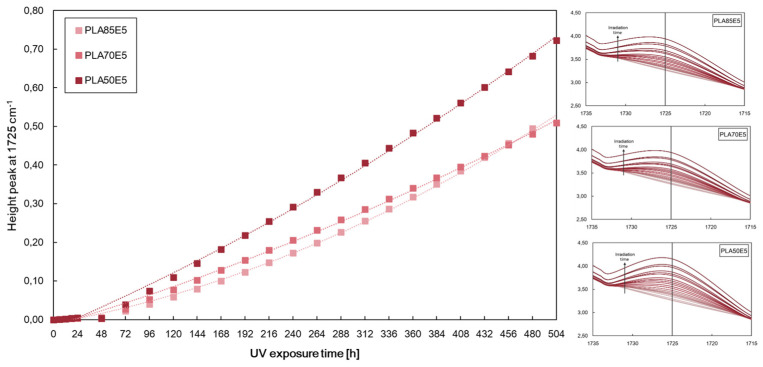
Height of peak at 1725 cm^−1^ as a function of the photo-oxidation time and selected zone in FTIR spectra of PLA/PA11/E blends at 85/15/5, 70/30/5, 50/50/5 wt%.

Figure 5 shows the FTIR spectra of the PLA/PA11/E blend (70/30/5 wt%) at different photo-oxidation times. The emergence of a shoulder up to peak, close to the large carbonyl peak, is clearly visible. Therefore, the peaks at 1845 cm^−1^ and 1725 cm^−1^, which change upon UVB exposure, have been chosen to monitor the photo-oxidation behaviour of the blends. Figure 6 and Figure 7 display the heights of peaks of PLA/PA11/E blends (85/15/5, 70/30/5, 50/50/5 % wt/wt) at 1845 cm^−1^ and 1725 cm^−1^, respectively, as a function of the irradiation times. As expected, the heights of both peaks increase with increasing exposure time for the PLA/PA11 blends. Interestingly, this increase is much more pronounced for samples with a high PA11 content, as follows 85/15/5 wt% < 70/30/5 wt% < 50/50/5 wt%. The latter suggests that the oxidation in both biopolymer phases becomes more pronounced by increasing the PA11 amount. These results are in agreement with the trends previously documented for PLA/PA11 blends without a compatibiliser [13]. It can be concluded that the presence of a compatibiliser does not have an explicit effect on the photo-oxidation resistance of PLA/PA11 biopolymer blends.

To sum up, the compatibiliser significantly reduced the size of the dispersed PA11 droplets in all PLA/PA11/E blends (85/15/5, 70/30/5, and 50/50/5 wt%), leading to improved processability, ductility and hydrophobicity through morphology refinement. However, it reduced the photo-oxidative stability of the blends, as evidenced by increased anhydride accumulation in the PLA phase and enhanced formation of oxygen-containing groups in the PA11 phase.

### 3.2. Effect of Compatibiliser on Biopolymer Blends Containing Metal Oxides

**Figure 8:**
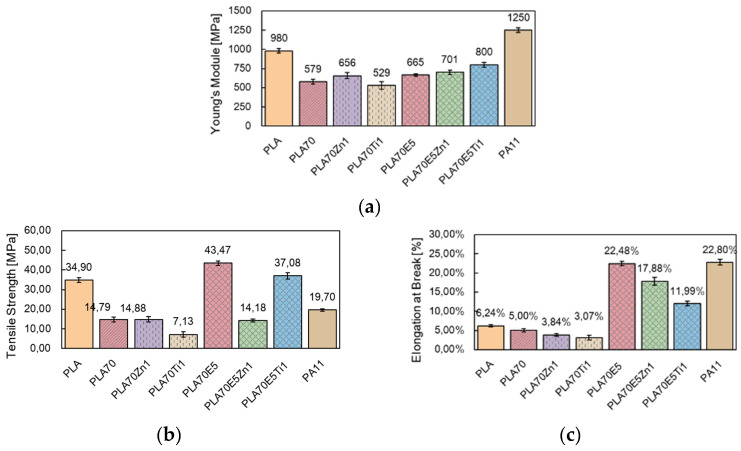
Mechanical properties: (**a**) Young’s modulus, E, (**b**) tensile strength, TS and (**c**) elongation at break, EB, of PLA/PA11 blends at 70/30 wt%, containing ZnO and TiO_2_ at 1 wt%, with and without compatibiliser agent (Elvaloy) at 5 wt%. Data on PLA/PA11 blends without compatibiliser have been published previously [13].

As demonstrated in a number of preceding studies, the PLA/PA11 blend at 70/30 wt% has been shown to exhibit optimal properties and performance [12,13,14]. Furthermore, the presence of metal oxides in the 70/30 wt% blend has been shown to have a beneficial effect. In light of the aforementioned points, the present study offers results pertaining to complex systems comprising PLA/PA11 (70/30 wt%) with incorporated ZnO and TiO_2_ (1 wt%), in conjunction with and without a compatibiliser (5 wt%). As demonstrated in Figure 8, the mechanical properties of all investigated systems are reported, and it is evident that the presence of a compatibiliser has a positive effect on both rigidity (i.e., Young’s modulus) and properties at break (i.e., tensile strength and elongation at break). The compatibiliser has been demonstrated to increase the Young’s modulus for the PLA/PA11/ZnO blend from 656 MPa to 701 MPa, thus indicating a beneficial effect. It is interesting to note that the compatibiliser has a more pronounced effect on the PLA/PA11/TiO_2_ blend, specifically resulting in an increase in the Young’s modulus value from 529 MPa to 800 MPa, suggesting the formation of a more resistant biobased system. It is important to note that both the elongation at break and the tensile strength of compatibilised biopolymer blends increase from three to four times in the presence of both metal oxides and a compatibiliser. This suggests that the compatibiliser has a significant beneficial effect, promoting chain slipping and making the materials ductile.

**Figure 9:**
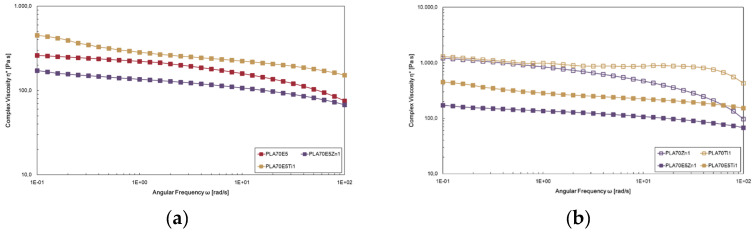
(**a**,**b**) Viscosity curves of PLA/PA11 blends at 70/30 wt%, containing ZnO and TiO_2_ at 1 wt%, with and without compatibiliser agent (Elvaloy) at 5 wt%. Data on PLA/PA11 blends without compatibiliser have been published previously [13].

Figure 9a,b illustrates the viscosity trends of PLA/PA11 blends at 70/30 wt% containing ZnO and TiO_2_ at 1 wt%, with and without a compatibiliser agent (Elvaloy) at 5 wt%. It is evident that the compatibiliser supports both ZnO and TiO_2_ particle dispersion, resulting in an increase in viscosity in comparison to the viscosity curve of the PLA/PA11/E blend (see Figure 9a). It is interesting to note that the viscosity trends of compatibilised blends containing both ZnO and TiO_2_ show lower values than those of uncompatibilised biopolymer blends, see Figure 9b. This is probably due to the fact that the compatibiliser supports the dispersion and chain orientation of the particles in the melt state, in a similar way to the solid state. (The Supplementary Figures S4 and S5 show the trends of storage, G′, and loss, G″, moduli.)

**Figure 10:**
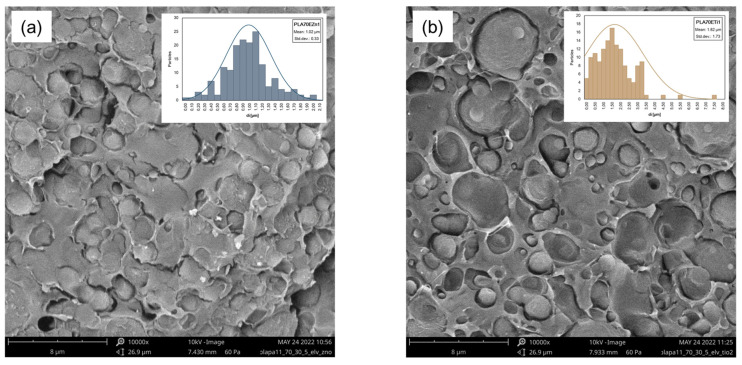
SEM images of PLA/PA11/E = 70/30/5 wt% blend containing 1 wt% of (**a**) ZnO, (**b**) TiO_2_.

**Figure 11:**
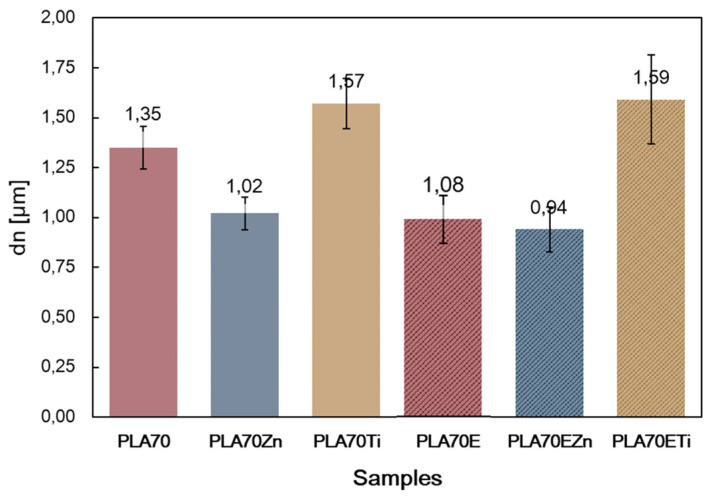
Numerical mean diameter for blends of PLA and PA11 in different concentrations and corresponding samples in the presence of Elvaloy PTW.

Morphology observations were performed using scanning electron microscopy (SEM), and Figure 10a,b shows representative SEM images of the PLA/PA11/E = 70/30/5 wt./wt. blend containing ZnO and TiO_2_ particles at 1 wt%. The inserts in Figure 10a,b illustrate the distribution of PA11 particle dimensions. Furthermore, the mean diameter (dn) of PA11 particles in PLA and the ratio (D) between dv and dn in PLA/PA11 are reported in Table 3 and Figure 11, respectively. Despite the limitations of the study, the impact of the compatibiliser on the reduction of PA11 droplet dimensions is discernible, indicating potential benefits in terms of enhancing the dispersion of metal oxides and a marginal decrease in PA11 droplet dimensions. The precise statistical analysis indicates that the compatibiliser at 5 wt%, despite its restricted efficacy, exerts a favourable influence on the overall morphology of the complex system.

Therefore, in accordance with the findings of preceding studies [13,14], it has been determined that clay and metal oxide particles are predominantly situated within the interphase between the two biopolymer phases. A minimal quantity of these particles is found within the more polar polymeric phase, such as PA11, at low concentrations, and this leads to a reduction in the dimensions of the droplet-dispersed phase. The findings of this study suggest that the presence of a compatibiliser, exhibiting a limited effect, can result in a reduction in the dimensions of the PA11 droplets without causing significant alterations to their morphology, provided that the morphology appears droplet-like.

**Figure 12:**
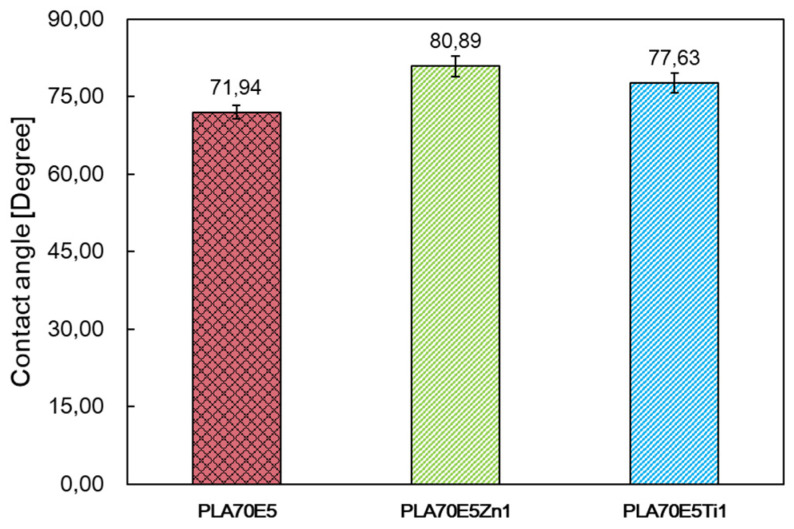
Water Contact Angle (WCA) of PLA/PA11/E = 70/30/5 wt% blend, also containing 1 wt% ZnO and 1 wt% TiO_2_.

The WCA values of the investigated biopolymer blends are displayed in Figure 12. As demonstrated in our previous study, the WCA of neat PLA/PA11 (70/30 wt%) was approximately 64.75°. It is evident that the incorporation of a compatibiliser results in the formation of a more hydrophobic material, as evidenced by the WCA value of approximately 71.94°. It is noteworthy that the presence of both a compatibiliser agent and ZnO and TiO_2_ particles results in a significant enhancement of the hydrophobicity of the blends; the WCA values of PLA/PA11/E blend containing ZnO and TiO_2_ are approximately 80.89° and ca. 77.63°, respectively. The latter phenomenon can be rationalised by considering the role of the compatibiliser, which has been demonstrated to facilitate the dispersion of metal oxide particles. This, in turn, hinders the penetration of water molecules, thereby contributing to the observed outcomes.

In this instance, it is noteworthy that the films have been processed under identical conditions, and the alterations in WCA values can be ascribed to the blend compositions, i.e., to the presence of the metal oxides together with the compatibiliser. However, the effect of external physical conditions has been minimised in order to emphasise the compositions rather than other factors.

**Figure 13:**
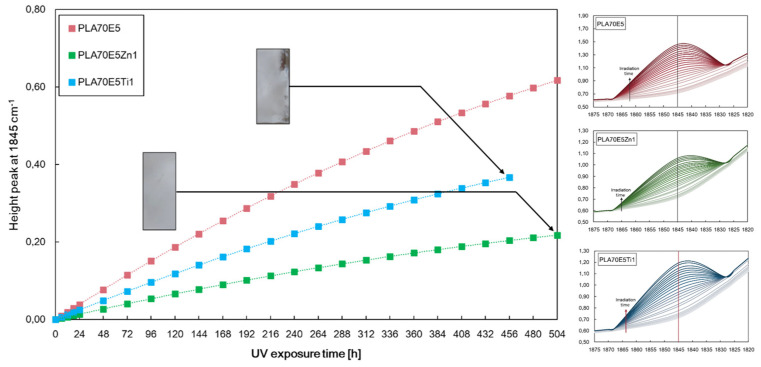
Height of peak at 1845 cm^−1^ as a function of the photo-oxidation time and selected zone in FTIR spectra of PLA/PA11/E blend at 70/30/5 wt% with and without ZnO and TiO_2_.

**Figure 14:**
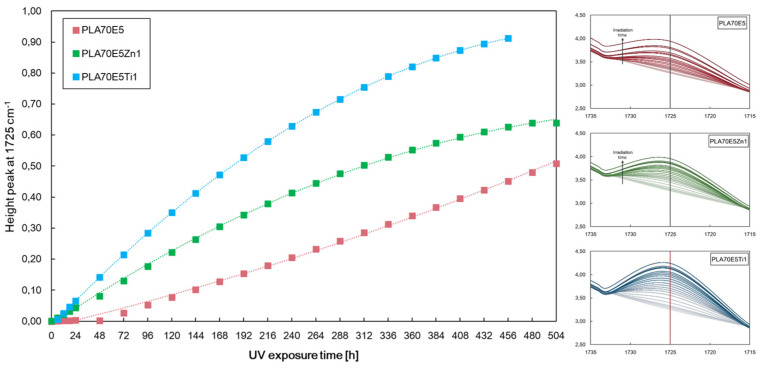
Height of peak at 1725 cm^−1^ as a function of the photo-oxidation time and selected zone in FTIR spectra of PLA/PA11/E blend at 70/30/5 wt% with and without ZnO and TiO_2_.

Figure 13 and Figure 14 show the peak heights at 1845 cm^−1^ and 1725 cm^−1^, which are related to the accumulation of PLA anhydride functionalities and oxygen-containing groups in the PA11 phase, as discussed above. The presence of both the compatibiliser and the metal oxides does not favour the accumulation of PLA anhydride functionalities, and this suggests that the metal oxides are not located selectively in the PLA phase. Conversely, the presence of both the compatibiliser and metal oxides promotes the formation of oxygen-containing groups in PA11, which confirms the selective location of metal oxides in this phase. According to our previous work, the presence of metal oxides accelerates the photoxidation of biopolymer matrices, and this process occurs quickly if the particles are well dispersed. Therefore, the analysis of photoxidation behaviour suggests that these complex systems, based on biopolymer blends and containing compatibiliser and metal oxides, are not useful for outdoor application, due to the fast accumulation of oxygen-containing groups.

Therefore, the combined addition of metal oxides (ZnO or TiO_2_) and a compatibiliser did not provide the expected synergistic effect. Increasing film hydrophobicity, particularly with ZnO, did not improve photo-oxidative stability, likely due to the interfacial localisation of the metal oxides. The resulting reduced durability may be advantageous for biodegradable packaging applications requiring accelerated degradation.

## 4. Conclusions

Biopolymer-based blends (i.e., PLA/PA11), with and without compatibiliser, such as ethylene butyl-acrylate glycidyl methacrylate (Elvaloy), and metal oxides, such as ZnO and TiO_2_, have been formulated successfully and subjected to accurate mechanical, rheological, morphological and thermal analysis.

The presence of a compatibiliser significantly reduces the dimensions of PA11 droplets, and this is clearly noticeable for all investigated ratios PLA/PA11/E, i.e., 85/15/5, 70/30/5 and 50/50/5 wt%. The presence of a compatibiliser plays a favourable role in overall mechanical behaviour, making the blends easier to process and more ductile. Furthermore, due to the presence of a compatibiliser agent, the formulated biopolymer films become more hydrophobic, also because of a morphology change. The presence of a compatibiliser leads to the formulation of biopolymer blends with reduced photo-oxidative resistance, considering that both anhydride accumulation in PLA and the formation of oxygen-containing groups in the PA11 phase increase significantly.

The presence of both metal oxides (ZnO and TiO_2_) and a compatibiliser, due to the formation of complex systems, does not have the expected beneficial effect. Regarding the dimension of PA1 dispersed droplets, the dimensions of the droplets are significantly influenced not only by the presence of the compatibiliser but also by the nature of the metal oxide. The biopolymer-based films containing both metal oxide and the compatibiliser result in a more hydrophobic surface, and this is more pronounced for the film with ZnO. Interestingly, the anhydride accumulation in PLA results slows down due to the presence of both metal oxide and the compatibiliser, although the formation of oxygen-containing groups increases, suggesting the absence of protection action of the metal oxides, probably due to their selective location at the interphase, rather than in the more polar phase. Therefore, the reduced durability and photo-oxidation behaviour of bio-blends could be considered to be favourable for the development of packaging solutions with a quick biodegradation time.

To sum up, the presence of a compatibiliser has been shown to exert a discernible favourable influence on the properties of PLA/PA11 blends. However, in PLA/PA11 blends containing metal oxides, the effect of the compatibiliser may be subject to considerable variability due to the complexity of the systems, and the identification of a unique trend for the properties is more complex.

## Figures and Tables

**Table 1:** Average diameter (dn) of PA11 particles in PLA and ratio (D) between dn and dv in PLA/PA11 blends with and without compatibiliser. Data on PLA/PA11 blends without compatibiliser have been published previously [13].

Samples	Σni	$d_{n}\;[\mu m]$	D
PLA/PA11 = 85/15 wt%	201	1.28 ± 0.10	3.46
PLA/PA11 = 70/30 wt%	166	1.35 ± 0.11	1.41
PLA/PA11 = 50/50 wt%	129	2.05 ± 0.12	1.65
PLA/PA11/E = 85/15/5 wt%	159	1.01 ± 0.12	0.93
PLA/PA11/E = 70/30/5 wt%	134	1.08 ± 0.13	1.08
PLA/PA11/E = 50/50/5 wt%	161	1.29 ± 0.15	1.19

**Table 2:** DSC data of neat PLA, PA11, PLA/PA11 = 70/30 wt% and PLA/PA11/E = 70/30/5 wt%.

Samples	Tg [°C]	Tc1 [°C]	Tc2 [°C]	Tm1 [°C]	Tm2 [°C]	Xc1 [%]	Xc2 [%]
PLA	62.23	96.18	-	174.54	-	14.59	-
PA11	-	-	146.27	190.10	-	-	
PLA/PA11/E = 70/30	61.78	97.04	147.16	167.76	183.29	8.92	2.71
PLA/PA11/E = 70/30/5	61.01	96.04	148.11	166.97	184.02	7.21	1.95

**Table 3:** Number of analysed particles (ni), average numerical diameter (dn) of PA11 particles in PLA and dispersion *D* = *d_v_*/*d_n_*.

Samples	Σni	$d_{n}\;[\mu m]$	D
PLA70E	134	1.08 ± 0.13	1.08
PLA70EZn	169	0.94 ± 0.11	0.94
PLA70ETi	141	1.59 ± 0.22	1.59

## Data Availability

The original contributions presented in this study are included in the article/Supplementary Material. Further inquiries can be directed to the corresponding author.

## Supplementary Materials

The following supporting information can be downloaded at: https://www.mdpi.com/article/10.3390/polym18141782/s1.

## References

[1] Kaplan D.L. (1998). Introduction to Biopolymers from Renewable Resources. Biopolymers from Renewable Resources.

[2] Pilla S. (2011). Handbook of Bioplastics and Biocomposites Engineering Applications.

[3] Hottle T.A., Bilec M.M., Landis A.E. (2013). Sustainability assessments of bio-based polymers. Polym. Degrad. Stab..

[4] Yates M.R., Barlow C.Y. (2013). Life cycle assessments of biodegradable, commercial biopolymers—A critical review. Resour. Conserv. Recycl..

[5] Kawasaki J., Silalertruksa T., Scheyvens H., Yamanoshita M. (2015). Environmental sustainability and climate benefits of green technology for bioethanol production in Thailand. J. Int. Soc. Southeast Asian Agric. Sci..

[6] Narancic T., Cerrone F., Beagan N., O’Connor K.E. (2020). Recent advances in bioplastics: Application and biodegradation. Polymers.

[7] George A., Sanjay M.R., Srisuk R., Parameswaranpillai J., Siengchin S. (2020). A comprehensive review on chemical properties and applications of biopolymers and their composites. Int. J. Biol. Macromol..

[8] Di Bartolo A., Infurna G., Dintcheva N.T. (2021). A review of bioplastics and their adoption in the circular economy. Polymers.

[9] Li K., Peng J., Turng L.-S., Huang H.-X. (2011). Dynamic rheological behavior and morphology of polylactide/poly(butylenes adipate-co-terephthalate) blends with various composition ratios. Adv. Polym. Technol..

[10] Stoclet G., Seguela R., Lefebvre J.-M. (2011). Morphology, thermal behavior and mechanical properties of binary blends of compatible biosourced polymers: Polylactide/polyamide11. Polymer.

[11] Patel R., Ruehle D.A., Dorgan J.R., Halley P., Martin D. (2013). Biorenewable blends of polyamide-11 and polylactide. Polym. Eng. Sci..

[12] Nuzzo A., Coiai S., Carroccio S., Dintcheva N.T., Gambarotti C., Filippone G. (2014). Heat-resistant fully bio-based nanocomposite blends based on Poly(lactic acid). Macromol. Mater. Eng..

[13] Morici E., Pecoraro G., Carroccio S.C., Bruno E., Scarfato P., Filippone G., Dintcheva N.T. (2024). Understanding the Effects of Adding Metal Oxides to Polylactic Acid and Polylactic Acid Blends on Mechanical and Rheological Behaviour, Wettability, and Photo-Oxidation Resistance. Polymers.

[14] Infurna G., Scamporrino A.A., Morici E., Bruno E., Pecoraro G., Dintcheva N.T. (2025). Performance and Durability of Biopolymer Blends Containing Modified Metal Oxide Particles. Polymers.

[15] Jim Jem K., Tan B. (2020). The development and challenges of poly(lactic acid) and poly(glycolic acid). Adv. Ind. Eng. Polym. Res..

[16] Bangar S.P., Whiteside W.S., Ashogbon A.O., Kumar M. (2021). Recent advances in thermoplastic starches for food packaging: A review. Food Packag. Shelf Life..

[17] Surendren A., Mohanty A.K., Liu Q., Misra M. (2022). A review of biodegradable thermoplastic starches, their blends and composites: Recent developments and opportunities for single-use plastic packaging alternatives. Green Chem..

[18] Laorenza Y., Harnkarnsujarit N. (2024). Surface adhesion and physical properties of modified TPS and PBAT multilayer film. Food Packag. Shelf Life.

[19] Reichert C.L., Bugnicourt E., Coltelli M.-B., Cinelli P., Lazzeri A., Canesi I., Braca F., Martínez B.M., Alonso R., Agostinis L. (2020). Bio-Based Packaging: Materials, Modifications, Industrial Applications and Sustainability. Polymers.

[20] Andrzejewski J., Das S., Lipik V., Mohanty A.K., Misra M., You X., Tan L.P., Chang B.P. (2024). The Development of Poly(lactic acid) (PLA)-Based Blends and Modification Strategies: Methods of Improving Key Properties towards Technical Applications—Review. Materials.

[21] Baranwal J., Barse B., Fais A., Delogu G.L., Kumar A. (2022). Biopolymer: A Sustainable Material for Food and Medical Applications. Polymers.

[22] Mahmood A., Patel D., Hickson B., DesRochers J., Hu X. (2022). Recent Progress in Biopolymer-Based Hydrogel Materials for Biomedical Applications. Int. J. Mol. Sci..

[23] Chai Q., Jiao Y., Yu X. (2017). Hydrogels for Biomedical Applications: Their Characteristics and the Mechanisms behind Them. Gels.

[24] Bealer E.J., Onissema-Karimu S., Rivera-Galletti A., Francis M., Wilkowski J., Salas-de la Cruz D., Hu X. (2020). Protein-Polysaccharide Composite Materials: Fabrication and Applications. Polymers.

[25] Sharma S., Sharma B., Manral A., Bajpai P.K., Jain P., Thomas S., Gopi S., Amalraj A. (2021). Biopolymers in the automobile and adhesive industries. Biopolymers and Their Industrial Applications: From Plant, Animal, and Marine Sources, to Functional Products.

[26] Udayakumar G.P., Subbulakshmi M., Selvaganesh B., Sivarajasekar N., Rambabu K., Sivamani S., Sivakumar N., Maran J.P., Hosseini-Bandegharaei A. (2021). Ecofriendly biopolymers and composites: Preparation and their applications in water-treatment. Biotechnol. Adv..

[27] Atiwesh G., Mikhael A., Parrish C.C., Banoub J., Le T.A.T. (2021). Environmental impact of bioplastic use: A review. Heliyon.

[28] Sonkaria S., Cho J., Jo H.S., Kim H.J., Thomas S., AR A., Jose Chirayil C., Thomas B. (2023). Biopolymer-Based Blends. Handbook of Biopolymers.

[29] Akinsemolu A.A., Idowu A.M., Onyeaka H.N. (2024). Recycling Technologies for Biopolymers: Current Challenges and Future Directions. Polymers.

[30] Hasan M.R., Davies I.J., Pramanik A., John M., Biswas W.K. (2025). Recycling Post-Consumed Polylactic Acid Waste Through Three-Dimensional Printing: Technical vs. Resource Efficiency Benefits. Sustainability.

[31] Javaid H., Khan M., Mustafa K., Musaddiq S. (2022). Biodegradable Plastics as a Solution to the Challenging Situation of Plastic Waste Management. Handbook of Biodegradable Materials.

[32] Sandhya P.K., Sreekala M.S., Thomas S., Thomas S., AR A., Jose Chirayil C., Thomas B. (2022). Biopolymer-Based Blend Nanocomposites. Handbook of Biopolymers.

[33] Battegazzore D., Alongi J., Frache A. (2014). Poly(lactic acid)-Based Composites Containing Natural Fillers: Thermal, Mechanical and Barrier Properties. J. Polym. Environ..

[34] Machado A.C., Costa A.F., Rodrigues Â.R., Moreira P.F., Duarte F.M., Pontes A.J. (2025). Waste Coffee Silver Skin as a Natural Filler in PLA-Based Filaments for Fused Filament Fabrication (FFF) Printing. Polymers.

[35] McKay I., Vargas J., Yang L., Felfel R.M. (2024). A Review of Natural Fibres and Biopolymer Composites: Progress, Limitations, and Enhancement Strategies. Materials.

[36] Kakroodi A.R., Kazemi Y., Nofar M., Park C.B. (2017). Tailoring poly(lactic acid) for packaging applications via the production of fully bio-based in situ microfibrillar composite films. Chem. Eng. J..

[37] Nofar M., Salehiyan R., Ciftci U., Jalali A., Durmuş A. (2020). Ductility improvements of PLA-based binary and ternary blends with controlled morphology using PBAT, PBSA, and nanoclay. Compos. Part B Eng..

[38] Puglisi R., Scamporrino A.A., Dintcheva N.T., Filippone G., Bruno E., Scarfato P., Cerruti P., Carroccio S.C. (2023). Photo- and Water-Degradation Phenomena of ZnO Bio-Blend Based on Poly(lactic acid) and Polyamide 11. Polymers.

[39] Le A.T., Pung S., Sreekantan S., Matsuda A., Huynh D.P. (2019). Mechanisms of removal of heavy metal ions by ZnO particles. Heliyon.

[40] Alardhi S.M., Abdalsalam A.H., Ati A.A., Abdulkareem M.H., Ramadhan A.A., Taki M.M., Abbas Z.Y. (2024). Fabrication of polyaniline/zinc oxide nanocomposites: Synthesis, characterization and adsorption of methylene orange. Polym. Bull..

[41] Pachaiappan R., Rajendran S., Loke Show P., Manavalan M., Naushad M. (2021). Metal/metal oxide nanocomposites for bactericidal effect: A review. Chemosphere.

[42] Yadav H.M., Kim J.-S., Pawar S.H. (2016). Developments in photocatalytic antibacterial activity of nano TiO_2_: A review. Korean J. Chem. Eng..

[43] Akira Fujishima A., Rao T.N., Tryk D.A. (2000). Titanium dioxide photocatalysis. J. Photochem. Photobiol. C Photochem. Rev..

[44] Zagloul H., Dhahri M., Bashal A.H., Khaleil M.M., Habeeb T.H., Khalil K.D. (2024). Multifunctional Ag_2_O/chitosan nanocomposites synthesized via sol-gel with enhanced antimicrobial, and antioxidant properties: A novel food packaging material. Int. J. Biol. Macromol..

[45] Mohammed H.A., Eddine L.S., Souhaila M., Hasan G.G., Kir I., Abdullah J.A.A. (2023). Green Synthesis of SnO_2_ Nanoparticles from Laurus nobilis L. Extract for Enhanced Gelatin-Based Films and CEF@SnO_2_ for Efficient Antibacterial Activity. Food Bioprocess Technol..

[46] Trabelsi A.B.G., Mostafa A.M., Alkallas F.H., Elsharkawy W.B., Al-Ahmadi A.N., Ahmed H.A., Nafee S.S., Pashameah R.A., Mwafy E.A. (2023). Effect of CuO Nanoparticles on the Optical, Structural, and Electrical Properties in the PMMA/PVDF Nanocomposite. Micromachines.

[47] Gvozdenko A.A., Siddiqui S.A., Blinov A.V., Golik A.B., Nagdalian A.A., Maglakelidze D.G., Statsenko E.N., Pirogov M.A., Blinova A.A., Sizonenko M.N. (2022). Synthesis of CuO nanoparticles stabilized with gelatin for potential use in food packaging applications. Sci. Rep..

[48] Mehrabi Mazidi M., Sharifi H., Razavi Aghjeh M.K., Zare L., Khonakdar H.A., Reuter U. (2023). Super-tough PLA-based blends with excellent stiffness and greatly improved thermal resistance via interphase engineering. ACS Appl. Mater. Interf..

[49] Fredi G., Dorigato A. (2024). Compatibilization of biopolymer blends: A review. Adv. Ind. Eng. Polym. Res..

[50] Khankrua R., Wiriya-Amornchai A., Triamnak N., Suttiruengwong S. (2023). Biopolymer blends based on poly(lactic acid) and polyamide for durable applications. Polym.-Plast. Technol. Mater..

[51] Gardette M., Thérias S., Gardette J.-L., Murariu M., Dubois P. (2011). Photooxidation of polylactide/calcium sulphate composites. Polym. Degrad. Stab..

[52] Pérez Amaro L., Cicogna F., Passaglia E., Morici E., Oberhauser W., Al-Malaika S., Dintcheva N.T., Coiai S. (2016). Ther-mo-oxidative stabilization of poly(lactic acid) with antioxidant intercalated layered double hydroxides. Polym. Degrad. Stab..

[53] Dintcheva N.T., Al-Malaika S., Morici E. (2015). Novel organo-modifier for thermally stable polymer-layered silicate nanocomposites. Polym. Degrad. Stab..

[54] Dehouche N., Kaci M., Focke W.W., Van Der Merwe L. (2022). Accelerated Photo-Oxidation of Polyamide 11 Nanocomposites under Various Clays Nanofillers. Macromol. Symp..

