# Supplementary material for: Effect of the Compatibiliser on the Poly(Lactic Acid)—Polyamide 11 Blends with and Without Metal Oxides: Properties, Performance and Durability
Source: Polymers (Basel). 2026 Jul 21;18(14):1782. doi: 10.3390/polym18141782 (PMC13419301; doi:10.3390/polym18141782)
Supplement: Supplementary file 1 [file polymers-18-01782-s001.zip › polymers-4345978-supplementary.pdf]

Supplementary information

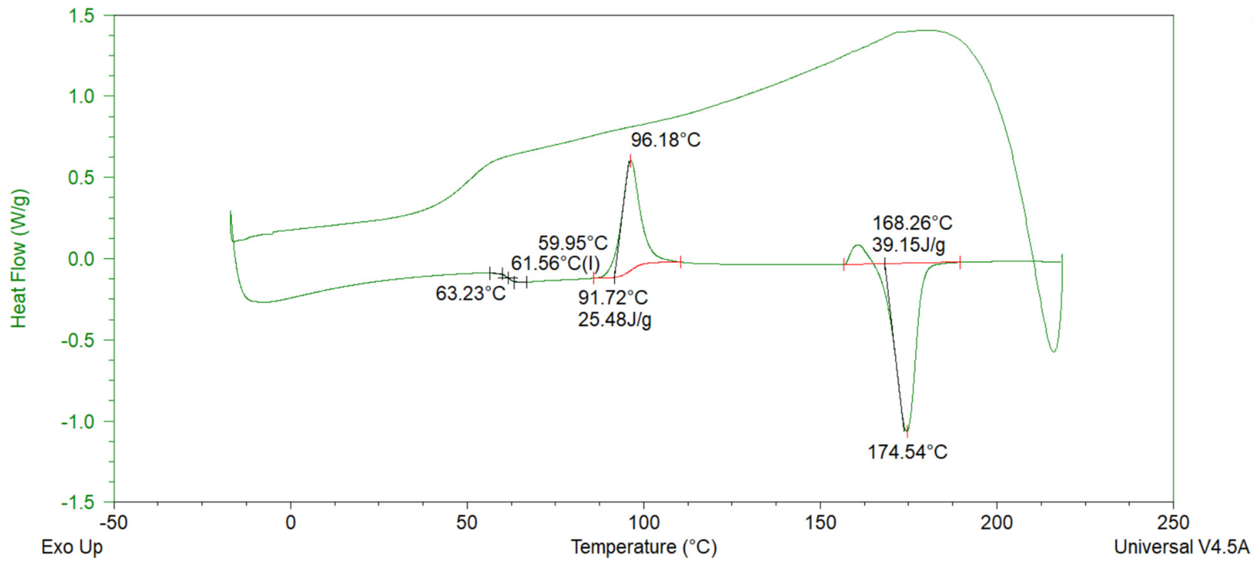

Figure S1. DSC traces of neat PLA.

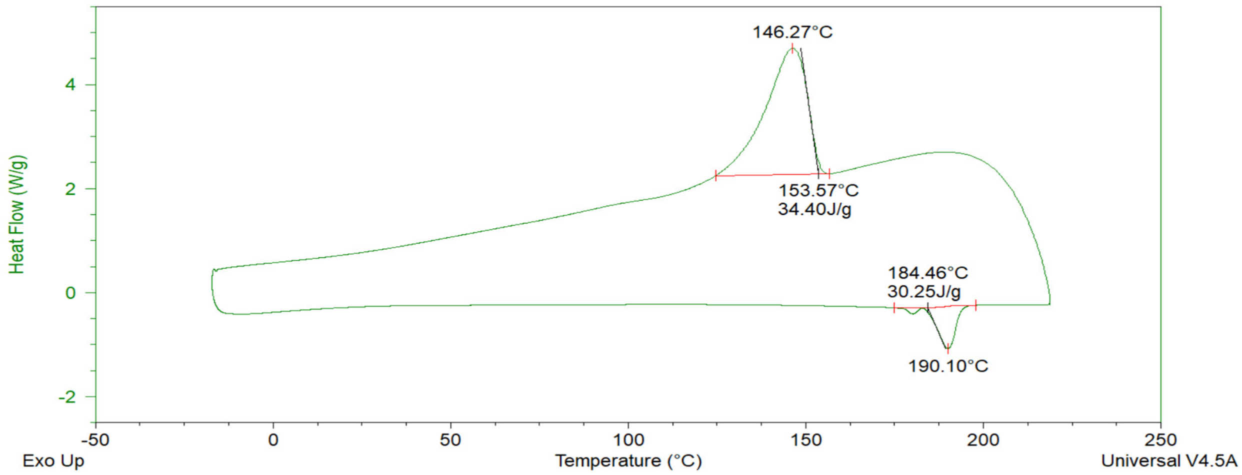

Figure S2. DSC traces of neat PA11.

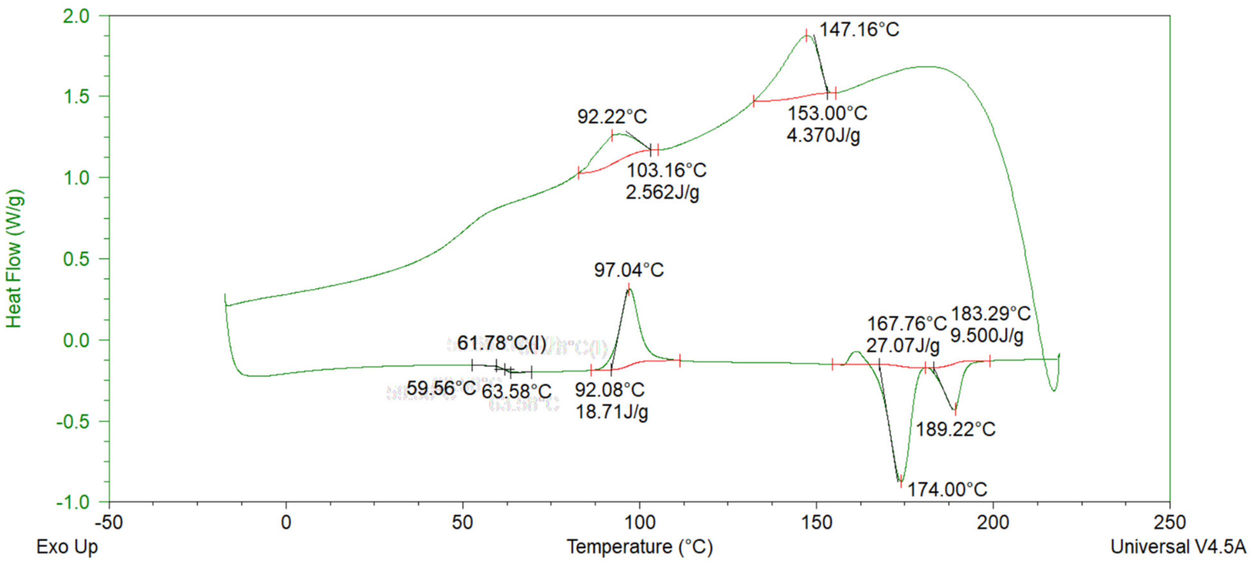

Figure S3. DSC traces of PLA/PA11/Elvaloy = 70/30/5 wt.%.

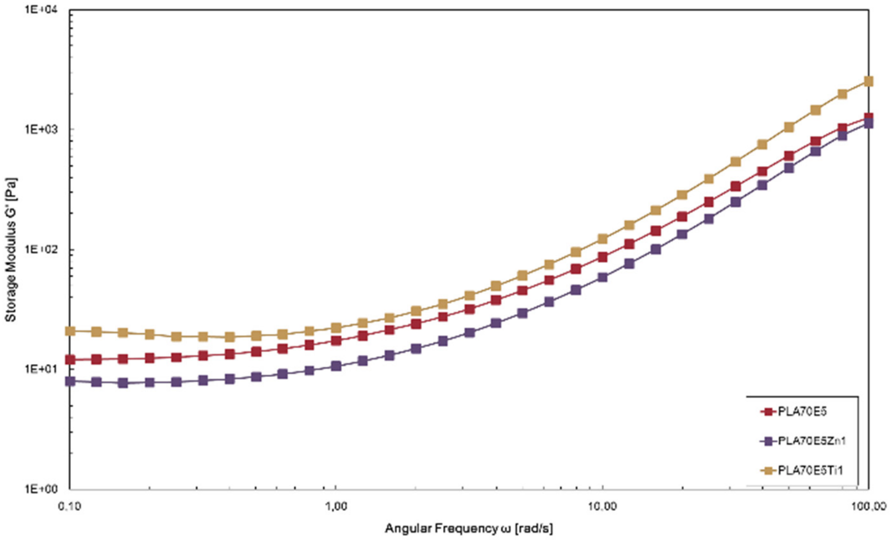

Figure S4. Storage modulus (G') of PLA/PA11/Elvaloy = 70/30/5 wt.% without and with Zn and TiO_2.

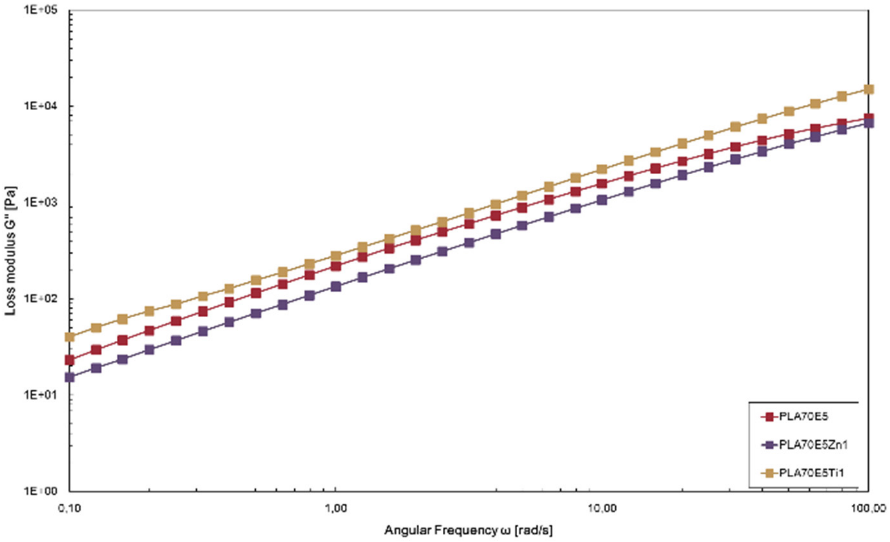

Figure S5. Loss modulus (G'') of PLA/PA11/Elvaloy = 70/30/5 wt.% without and with Zn and TiO_2.
